# D-DSC: Decoding Delay-based Distributed Source Coding for Internet of Sensing Things

**DOI:** 10.1371/journal.pone.0193154

**Published:** 2018-03-14

**Authors:** Metin Aktas, Murat Kuscu, Ergin Dinc, Ozgur B. Akan

**Affiliations:** 1 Aselsan, Inc., 06370, Ankara, Turkey; 2 Department of Electrical and Electronics Engineering, Koc University, 34450, Istanbul, Turkey; 3 Electrical Engineering Division, Department of Engineering, University of Cambridge, CB3 0FA, Cambridge, United Kingdom; University of Texas at San Antonio, UNITED STATES

## Abstract

Spatial correlation between densely deployed sensor nodes in a wireless sensor network (WSN) can be exploited to reduce the power consumption through a proper source coding mechanism such as distributed source coding (DSC). In this paper, we propose the Decoding Delay-based Distributed Source Coding (D-DSC) to improve the energy efficiency of the classical DSC by employing the *decoding delay concept* which enables the use of the maximum correlated portion of sensor samples during the event estimation. In D-DSC, network is partitioned into clusters, where the clusterheads communicate their uncompressed samples carrying the side information, and the cluster members send their compressed samples. Sink performs joint decoding of the compressed and uncompressed samples and then reconstructs the event signal using the decoded sensor readings. Based on the observed degree of the correlation among sensor samples, the sink dynamically updates and broadcasts the varying compression rates back to the sensor nodes. Simulation results for the performance evaluation reveal that D-DSC can achieve reliable and energy-efficient event communication and estimation for practical signal detection/estimation applications having massive number of sensors towards the realization of Internet of Sensing Things (IoST).

## Introduction

Internet of Things (IoT), which defines the seamless interconnection and autonomous coordination of massive number of sensing and computing elements and physical entities through the Internet infrastructure, is an emerging research direction towards the long-standing goal of enabling the connected Universe. WSNs have evolved into Internet of Sensing Things (IoST) as part of the Internet of Things (IoT) [1], and now stand as one of the driving forces for the IoT framework [2]. The most critical challenge in IoST applications is the power limits as it is not practical to recharge the batteries of massive number of devices. Power consumption can be divided into three parts: sensing, communication, and data processing. The power usage of a sensor node is dominated by communication: transmission and reception [3]. Therefore, the lifetime of the sensor nodes is highly related with the amount of information to be communicated as throughly investigated in [4].

There have been several approaches to overcome the power limitations and increase the lifetime of IoST applications, such as the recently introduced framework of energy harvesting Internet of Things [5, 6], and ultra-low power sensor networks exploiting the receive diversity with spatially separated antennas [7–9]. In this paper, we focus on data compression with the motivation to reduce the redundancy in the information. Due to dense deployment of network nodes, IoST applications operate with spatial redundancy [10–13]. The spatial redundancy can be exploited by source coding. In this way, a node can reduce the number of bits to be transmitted with no or very small loss of the information. Recently, the source coding is dominated by *Distributed Source Coding* (DSC) because it only requires the correlation between the sensors for the compression [10, 11, 13–18]. The DSC is especially promising for sources with high correlation; this is always the case for densely employed sensor networks [19].

DSC can be defined as the compression of multiple correlated samples of sensors that *do not communicate with each other* and joint decoding of these compressed samples at the central point (sink in our case) [13]. DSC is first introduced by Slepian and Wolf [20] theoretically showing that separate encoding (with increased complexity at the joint decoder) is as efficient as joint encoding for lossless compression. Similar results were obtained by Wyner and Ziv [21] with regard to lossy coding of jointly Gaussian sources.

In recent years, the basics of DSC concept are carried out in WSNs. However, most of these works [11, 13–16, 18] only deal with data compression algorithms of spatially distributed sensor samples, in which a correlation model between sensor samples is used. As a correlation model, some predefined constant models [22] are used regardless of sensor data, which is not realistic for many practical WSN applications. [10] uses dynamic correlation model in which data compression and correlation update algorithms are introduced for small size networks, and not applicable for large size networks. Moreover, it is incomplete to be used in practical WSN applications.

Some practical usages of DSC in WSNs are introduced in [23–30]. DSC is used for data compression of low frequency signal components in [24]; and for the effective usage of DSC in practical WSN applications, energy efficient transmission protocol [23] and routing optimization [25] are introduced. [17] utilizes the syndromes to perform DSC, but it does not support varying-rate applications. These works do not effectively exploit the DSC in data compression. DSC compression performance is optimized by applying DSC sequentially among sensor nodes in [26] and [27]. Although all these algorithms try to maximize the achievable compression rate for DSC, their performance highly depends on sensor deployment and works well especially for large node density distributions. Therefore, a complete and unified communication solution exploiting the potential advantages of DSC and inherent correlation characteristics of WSN is yet to be developed. This reason is the main motivation for us to introduce D-DSC.

In this paper, we present complete and unified solution, *Decoding Delay-based Distributed Source Coding, D-DSC*, which incorporates DSC and decoding delay concept in WSN. D-DSC improves the encoding and joint decoding concepts of classical DSC, and it significantly reduces the power consumption of the nodes by maximizing the compression rates (*Υ*) by exploiting *propagation delay* between sensor readings. For the topology independent applications, D-DSC employs a self-organizing cluster-based structure and correlation tracking method for adaptation to any changes in network. D-DSC also incorporates the reliability control and retransmission mechanism to achieve the desired event distortion bound (*D*_*max*_). Salient features of D-DSC are as follows.

***Maximum Compression***—Sensor nodes receive the same source signal with different propagation delays and attenuations as explained in Section 2.2. As the delay increases, the similarity between sensor samples decreases and it results in a decrease in compression rate. To this end, D-DSC increases the correlation between sensor readings by delaying or advancing some sensor node data in transmission. In this way, D-DSC maximizes the correlation and compression rate at the expense of increasing latency. Since DSC only exploits the correlation between the raw sensor measurements, it is expected that significant improvements can be provided in delay tolerant applications by utilizing D-DSC. We also introduce an efficient joint decoder to reconstruct the maximally compressed data at the sink. Details of D-DSC compression algorithm and joint decoder are given in Section 2.2 and 3.2, respectively.

***Self-organizing***—Since the failure of nodes and change in the topology is highly probable in WSNs, D-DSC is design to have self-organizing capabilities. Therefore, D-DSC learns about the topology and organizes the network into clusters by using the correlation between sensor samples. In this way, D-DSC can be used in any WSN application regardless of network topology. Details of self-organizing feature and clustering algorithm are given in Section 3.1.

***Adaptive***—The network topology may change frequently due to malfunctioning nodes or the mobility of the event. To monitor these changes and avoid the performance degradation, D-DSC continuously updates the correlation estimate between sensor samples and reorganizes the clusters in order to adapt the topology changes, as explained in Section 3.2.1.

***Reliability Control***—D-DSC maximally compresses data in sensor nodes and reduces the redundancy in sensor readings. Thus, any packet loss will have significant impact on the accuracy of the vent estimation. To this end, D-DSC continuously monitors the packet loss rate and event distortion (*D*_*e*_) to request retransmissions from appropriate sensor nodes. D-DSC determines the contribution of all sensor nodes on the event estimation and find the minimum number of retransmissions required to achieve the desired event distortion bound (*D*_*max*_) as discussed in Section 3.2.2.

***Lightweight Encoder***—The complexity of the encoder have important effects on the cost of the application. Therefore, D-DSC adopts a simple tree-based encoder as in [10]. Therefore, D-DSC is applicable for any sensor node used in WSN applications.

The remainder of the paper is organized as follows. The basic principles and architecture of D-DSC are specified in Section 1. A correlation model identifying the relation between optimum compression rate and spatial correlation between sensor nodes is derived in Section 2. The reliability mechanism adopted by D-DSC, the notion of decoding delay, and algorithm operations are given in Section 3. Performance analysis and simulation results are presented in Section 4. Lastly, the conclusions are presented in Section 5.

## 1 Sensor data model of D-DSC and network architecture

In this paper, we assume that there are *M* omni-directional sensor nodes which are densely deployed and *L* point sources which are located randomly. In this model, sensor nodes receive the source signals with different propagation delays and magnitudes, varying according to the distance between the source and the sensor node. In addition, there is a random noise on the observed signal at each sensor. Therefore, data observed at each sensor node are represented as

$$\begin{array}{c} x_{m}\left(n\right)=\sum_{l=1}^{L}e^{-\lambda r_{m,l}}s_{l}\left(n-\frac{r_{m,l}}{v_{s}}\right)+w\left(n\right),\begin{array}{c} 1\leq m\leq M,\\ 1\leq l\leq L \end{array}, \end{array}$$ (1)

where *λ* is the attenuation coefficient, *r*_*m*,*l*_ is the distance between sensor node *m* and *l*^*th*^ event source, and *v*_*s*_ is the propagation speed of the source signals. *s*_*l*_(*n*), *x*_*m*_(*n*) and *w*(*n*) are the *l*^*th*^ source data, received data at the sensor node *i*, and noise at discrete time *n*, respectively. It is assumed that the sensor samples, *x*_*m*_(*n*), 1 ≤ *m* ≤ *M* are statistically stationary in time and zero-mean random processes.

There is one sink which controls the sensor nodes and processes the received sensor samples. In D-DSC, one of the sensor nodes, called master node which sends its raw (uncompressed) data, while the other nodes send their compressed data to the sink. From the DSC point of view, master node data is utilized as a *side information* at the joint decoder at the sink. The encoding bit numbers are determined by the sink and notified to all nodes periodically. Therefore, all the sensor nodes send their data in different bit numbers according to their correlation with the master node.

## 2 Data compression

In this section, data compression algorithm based on Distributed Source Coding (DSC) is explained and the propagation delay concept which maximizes the correlation between sensor nodes is introduced.

### 2.1 Distributed Source Coding

DSC is based on compression of one sensor samples by using the other correlated sensor samples. Assume that samples of sensor node *j* are received correctly at the sink. Then, sending the differences between samples of sensor nodes *i* and *j* suffices to correctly reconstruct the samples of sensor node *i*. Instead of sending the original samples of sensor node *i*, sending the differences saves the transmitted bit number. On the other hand, the communication between sensor nodes are restricted in WSNs due to power limitations. Theoretical results in [20] and [21] show that the compression can be performed without knowing the samples of the other sensor data by utilizing just the correlation between them. In this way, the decoder can predict the sensor data by utilizing the side information from the master node and compressed data from the slave nodes.

#### 2.1.1 Encoding

**Fig 1:**
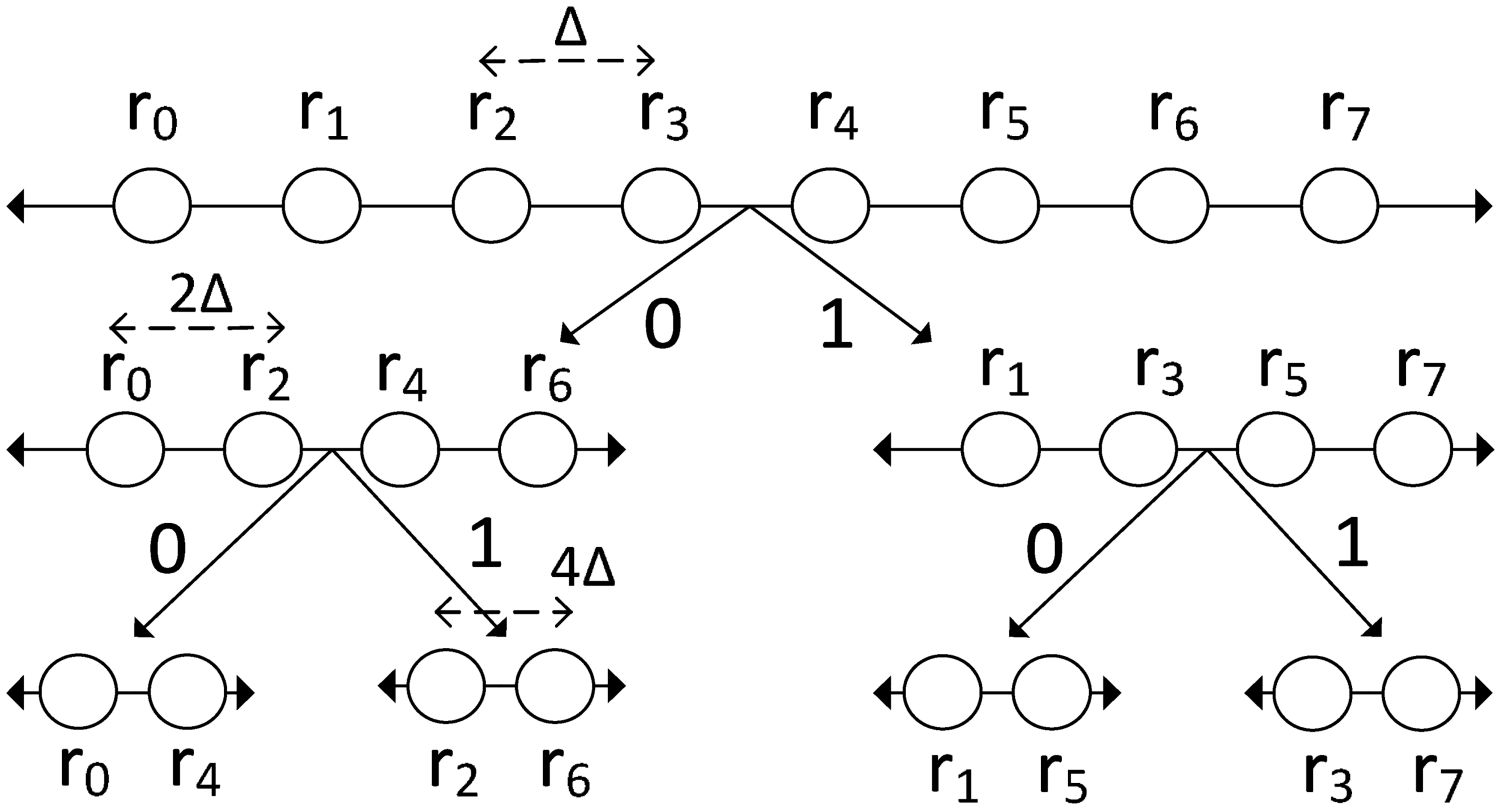
A tree based compression.

For simplicity, the encoding process is realized by using a tree-based encoder [10] in which the root-codebook and the sub-codebooks are used as in Fig 1. Sub-codebooks are determined by partitioning the root-codebook into two subsets consisting of even indexed ones and odd indexed ones. Repeating this process *n* times yields *n*-level tree structure that contains 2^*n*^ sub-codebooks. Samples of master nodes are encoded simply by finding the closest representation of the data from the 2^*n*_*u*_^ values in the root-codebook (this is typically done by an A/D converter). For encoding process of slave nodes, the encoder first finds the closest representation of the data from the 2^*n*_*u*_^ values in the root-codebook and then determines the sub-codebook that samples of slave node belongs to at level-*n*, where *n* is the minimum number of bits for encoding slave node data. The path through the root-codebook to this sub-codebook specifies the bits that are transferred to the sink [10].

#### 2.1.2 Joint decoding

To be compatible with the encoders used in sensor nodes, the sink uses tree-based decoder [10] in which the samples of master node are decoded simply by converting the received *n*_*u*_-bit value in the root-codebook to the data (this is typically done by a D/A converter). The decoder receives *n*_*i*,*j*_-bit value, *f*(*n*_*i*,*j*_), from the encoder of node *i* in cluster *j*. The decoder traverse the tree starting with the least significant bit (LSB) of *f*(*n*_*i*,*j*_) to determine the appropriate sub-codebook *C*(*i*) to use. The decoder then selects the value from *C*(*i*) which is the closest to the side information (samples of master node) and converts this value to the data [10]. If the side information is less than △_*sc*_/2 away from the samples of slave nodes, no decoding error occurs. △_*sc*_ is the spacing between the representative code values in the sub-codebook [10] which is represented as

$$\begin{array}{c} {△}_{sc}=2^{n_{i,j}^{tr}}△, \end{array}$$ (2)

where $n_{i,j}^{tr}$ is the minimum number of bits for encoding of slave nodes without decoding error in tree-based encoder and △ is the spacing between representative code values in the root-codebook [10]. Then, $n_{i,j}^{tr}$ can be found using the relation

$$\begin{array}{ccc} \frac{{△}_{sc}}{2} & \geq & |e_{i,j}\left(n\right)|,\\ 2^{n_{i,j}^{tr}-1}△ & \geq & |e_{i,j}\left(n\right)|, \end{array}$$ (3)

where *e*_*i*,*j*_(*n*) is the difference between samples of sensor nodes *i* and *j*. As stated in [10], $n_{i,j}^{tr}$ is found from (3) as,

$$\begin{array}{c} n_{i,j}^{tr}=\frac{1}{2}log_{2}(\frac{\sigma_{e_{i,j}}^{2}}{{△}^{2}P_{e}})+1, \end{array}$$ (4)

where $\sigma_{e_{i,j}}^{2}$ is the variance of *e*_*i*,*j*_(*n*), and *P*_*e*_ is the maximum probability error that *e*_*i*,*j*_(*n*) is greater than △_*sc*_, i.e., $P[|e_{i,j}\left(n\right)|>2^{n_{i,j}^{tr}-1}△]\leq P_{e}$.

### 2.2 Maximum-correlation based Distributed Source Coding

The minimum number of bits for encoding of slave nodes can be obtained by minimizing the variance of the difference between sensor samples and the side information, $\sigma_{e_{i,j}}^{2}$ as in (4). To this end, the minimum difference between samples of sensor node *i* and *j* for the data model that we utilized (1), can be defined as

$$\begin{array}{c} e_{i,j}\left(n\right)=x_{i}\left(n\right)-\xi x_{j}\left(n-m\right), \end{array}$$ (5)

where *x*_*j*_(*n* − *m*) is the delayed version of samples of node *j* by *m* samples, and *ξ* is a real positive value that is used to equalize the signal power in node *j* to in node *i*, which may be different due to the attenuation in propagation (*λ*) in (1). For the real valued samples, the minimum error in Mean-Square Error (MSE) sense can be found by minimizing (5) with respect to both *ξ* and *m* as

$$\begin{array}{c} e_{i,j}\left(n\right)=x_{i}\left(n\right)-\frac{R_{i,j}\left(d_{i,j}\right)}{\sigma_{j}^{2}}x_{j}\left(n-d_{i,j}\right), \end{array}$$ (6)

and the minimum variance of the sample difference is found as

$$\begin{array}{c} \sigma_{e_{i,j}}^{2}=\sigma_{i}^{2}-\frac{R_{i,j}^{2}\left(d_{i,j}\right)}{\sigma_{j}^{2}}, \end{array}$$ (7)

where $\sigma_{i}^{2}$ and $\sigma_{j}^{2}$ are the variances of samples of node *i* and *j*, respectively. *R*_*i*,*j*_(*m*) is the correlation function. The derivation of (6) and (7) is given in S1 Appendix. *d*_*i*,*j*_ is the sample difference between the maximum correlated data portion of node *i* and node *j*, which is defined as

$$\begin{array}{c} d_{i,j}=\arg\max_{m}\left\{R_{i,j}\left(m\right)\right\}, \end{array}$$ (8)

If we assume *x*_*i*_(*n*) and *x*_*j*_(*n*) are jointly ergodic processes, correlation function can be estimated as

$$\begin{array}{c} R_{i,j}\left(m\right)\approx \sum_{n=0}^{N-1}x_{i}^{*}\left(n-m\right)x_{j}\left(n\right), \end{array}$$ (9)

where *N* is the number of samples used in correlation estimation.

As in (6) and (7), D-DSC finds the maximum correlated data portion of sensor samples to minimize the sample differences. As the sample differences are minimized, D-DSC requires less number of bits for encoding slave node samples. The required minimum number of bits for encoding the samples of node *i* when samples of node *j* are used as the side information in the sink can be found for D-DSC by substituting (7) into (4), i.e.,

$$\begin{array}{c} n_{i,j}^{D-DSC}=\frac{1}{2}\log_{2}(\frac{\sigma_{i}^{2}-\frac{R_{i,j}^{2}\left(d_{i,j}\right)}{\sigma_{j}^{2}}}{{△}^{2}P_{e}})+1=\log_{2}(\frac{\sigma_{i}\sqrt{1-\rho_{i,j}^{2}}}{△\sqrt{P_{e}}})+1, \end{array}$$ (10)

where *ρ*_*i*,*j*_ is the correlation coefficient expressed as

$$\begin{array}{c} \rho_{i,j}=\frac{R_{i,j}\left(d_{i,j}\right)}{\sigma_{i}\sigma_{j}}. \end{array}$$ (11)

## 3 D-DSC protocol operation

This section explains the operational phases of D-DSC protocol including cluster organization, separate encoding and joint decoding processes, as well as network adaptation. In D-DSC, sensor nodes are responsible for only *encoding* operation. On the other hand, the sink performs three main components of D-DSC, namely *virtual clustering, joint decoding and network adaptation*, each of which is defined next in detail. The pseudocodes describing the operation of the sink are given in Algorithms 1 and 2 at the end of this section.

### 3.1 Virtual clustering

The attenuation of the event signals (*λ*) and the noise on the sensor outputs result in a significant decrease of the correlation between the sensor samples with increasing distance between the sensor nodes. It can be also revealed from (10) that the compression rate is exponentially degraded with decreasing correlation. Therefore, compressing the data of all the sensor nodes based on the side information of a single master node does not seem to be rational, especially for large networks. Hence, minimizing power consumption requires organizing the network into clusters to use multiple master nodes for data compression.

D-DSC is a self-organizing protocol, and thus, at initialization phase it has to learn the network topology and determine the energy-efficient clustering. In this phase, the sink requests all the sensor nodes for their raw sensing data, and computes the correlation coefficients as in (11) for each pair of sensor nodes (*i*, *j*).

D-DSC uses a *virtual clustering* in which only the sink knows about the node type, i.e., (*master* or *slave*). Sensor nodes only know the number of encoding bits (*n*_*i*,*j*_). Virtual clustering is performed by controlling two parameters, *ρ*_*i*,*j*_ and *d*_*i*,*j*_, depending on whether or not they satisfy the following clustering conditions

$$\begin{array}{c} \begin{array}{c} \rho_{i,j}\geq \rho_{th},\\ |d_{i,j}|\leq d_{th}f_{s}, \end{array} \end{array}$$ (12)

where *ρ*_*th*_ is the correlation coefficient threshold determined by the sink, *d*_*th*_ is the delay bound or application-specific deadline in seconds referring to the maximum allowable latency in communication, and *f*_*s*_ is the sampling frequency of the sensor samples.

The initial clustering is performed as follows. Among the nodes that do not belong to any cluster, the sink determines the master node *j* which is correlated with the largest set of remaining nodes satisfying the clustering conditions in (12). Node *j* is assigned as the master node, and the sensors in its correlated set are assigned as the slave nodes of cluster *j*.

Increasing correlation threshold (*ρ*_*th*_) seems to be an effective way of increasing compression rate. However, this may not be always true, as the number of clusters (*K*) and master nodes increases with increasing (*ρ*_*th*_). This amplifies the power consumption in the entire network as the master nodes are always required to send their uncompressed data to the sink. Thus, *ρ*_*th*_ should be selected optimally by solving the following inequality constraint optimization problem,

$$\begin{array}{c} \begin{array}{cc} \text{minimize} & E_{tot}=E\sum_{j=1}^{K}\left[\sum_{i=1}^{M_{j}-1}n_{i,j}+n_{u}\right],\\ \text{subject}\;\text{to} & \rho_{1}^{*}\leq \rho_{th}\leq \rho_{2}^{*}, \end{array} \end{array}$$

where *n*_*u*_ is the number of bits used for uncoded data transmission (*side information*), *n*_*i*,*j*_ is the number of bits used by node *i* in cluster *j* for encoding its raw data (10), and $\rho_{1}^{*}$ and $\rho_{2}^{*}$ are positive real numbers which depend on the system parameters. *K* and *M*_*j*_ are the number of clusters and the number of sensor nodes including master node in cluster *j*, respectively. *E* is the consumed power for one bit transmission. Since both *K* and *M*_*j*_ are functions of *ρ*_*th*_ and *d*_*th*_, the optimization problem given in (13) is difficult to solve analytically. The following assumptions simplify the problem:

1. All cross-correlation functions (9) are modeled as exponential functions:
  $$\begin{array}{c} R_{i,j}\left(m\right)=\sigma_{i}\sigma_{j}e^{-\frac{{\left(m-2d_{i,j}\right)}^{2}+m^{2}}{2\sigma }}, \end{array}$$ (13)
  where *σ*_*i*_ and *σ*_*j*_ are the standard deviation of samples of node *i* and *j*, respectively, and *σ* is the shape factor of the cross correlation function that is a real positive number. Larger *σ* corresponds to more correlated samples. In this model, the correlation takes its maximum value at *m* = *d*_*i*,*j*_ as in (8) and decreases exponentially as the time difference between sensor readings increases. This is reasonable, since due to the attenuation, the signal strength at the sensor output decreases as the sensor node goes away from the source signal and a measurement noise reduces the correlation between sensor outputs.
2. Sensor nodes encode data with the same number of bits obtained by (10) for *ρ*_*i*,*j*_ = *ρ*_*th*_ ∀*i*,*j*.
3. Sensor nodes are uniformly deployed on the event area with the size of [*r*_*e*_, *r*_*e*_] meters.

Based on these assumptions, the inequality constraint optimization problem in (13) can be rewritten in a more compact form as (see the derivation of (14) in S1 Appendix).

$$\begin{array}{cc} \text{minimize}\;E_{tot} & ≅EMlog_{2}(\left(2^{n_{u}}-1\right)\sqrt{1-\rho_{th}^{2}}+1)-\\ E\frac{f_{s}^{2}r_{e}^{2}}{\sigma \ln\rho_{th}v_{s}^{2}} & [n_{u}-log_{2}(\left(2^{n_{u}}-1\right)\sqrt{1-\rho_{th}^{2}}+1)],\\ \text{subject}\;\text{to} & e^{-\frac{f_{s}^{2}r_{e}^{2}}{\sigma v_{s}^{2}}}\leq \rho_{th}\leq e^{-\frac{f_{s}^{2}r_{e}^{2}}{\sigma v_{s}^{2}M}}, \end{array}$$ (14)

where *M* is the number of sensor nodes in the event area, and *v*_*s*_ is the propagation speed of the event source.

**Fig 2:**
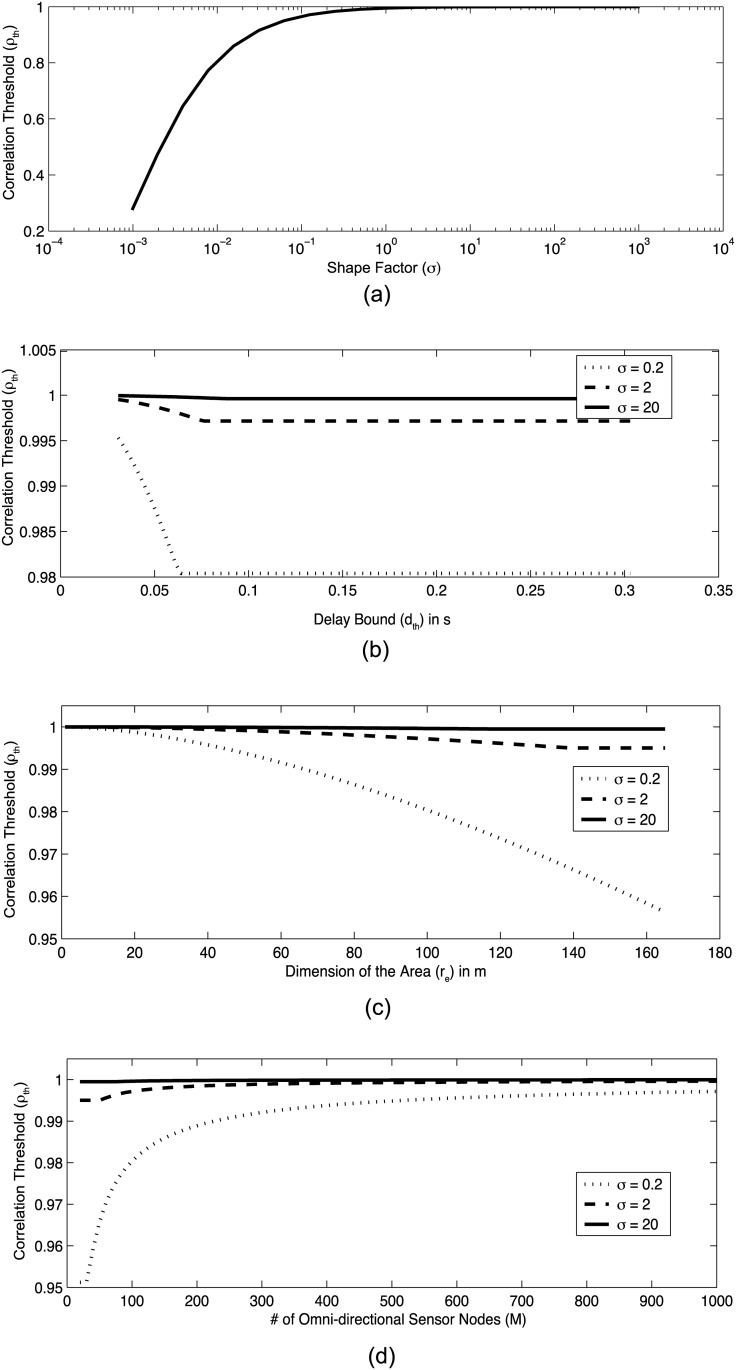
Optimum *ρ*_*th*_ for different (a) *σ*, (b) *d*_*th*_ and *σ*, (c) *r*_*e*_ m and *σ* (d) *M* and *σ*. The constant parameters for each figure are selected as, *M* = 100, *n*_*u*_ = 12bits, *r*_*e*_ = 100m, *v*_*s*_ = 330m/s and *d*_*th*_ = 0.1s.

Since this inequality constraint optimization problem cannot be solved analytically, we numerically solve it using MATLAB, and the resultant optimum *ρ*_*th*_ for varying parameters is illustrated in Fig 2.

Once the optimum clustering that satisfies (12) is performed, the sink asks for an extra uncompressed data with the length of *l*_*ex*_, i.e., *excess information*, from each master node to use at the joint decoder as in Section 2.2. The length of an excess information is related with the delay threshold by

$$\begin{array}{c} l_{ex}=d_{th}f_{s}, \end{array}$$ (15)

While the master nodes send an excess information to the sink, slave nodes store their raw data but do not transmit. After collecting the excess information from each master node, the sink informs all the sensor nodes about the number of encoding bits, *n*_*i*,*j*_ (10), and each sensor node transmits their data coded with it. Slave nodes transmit their coded data starting from their internal caches, which results *l*_*ex*_ samples delay in transmission.

### 3.2 Joint decoding

**Fig 3:**
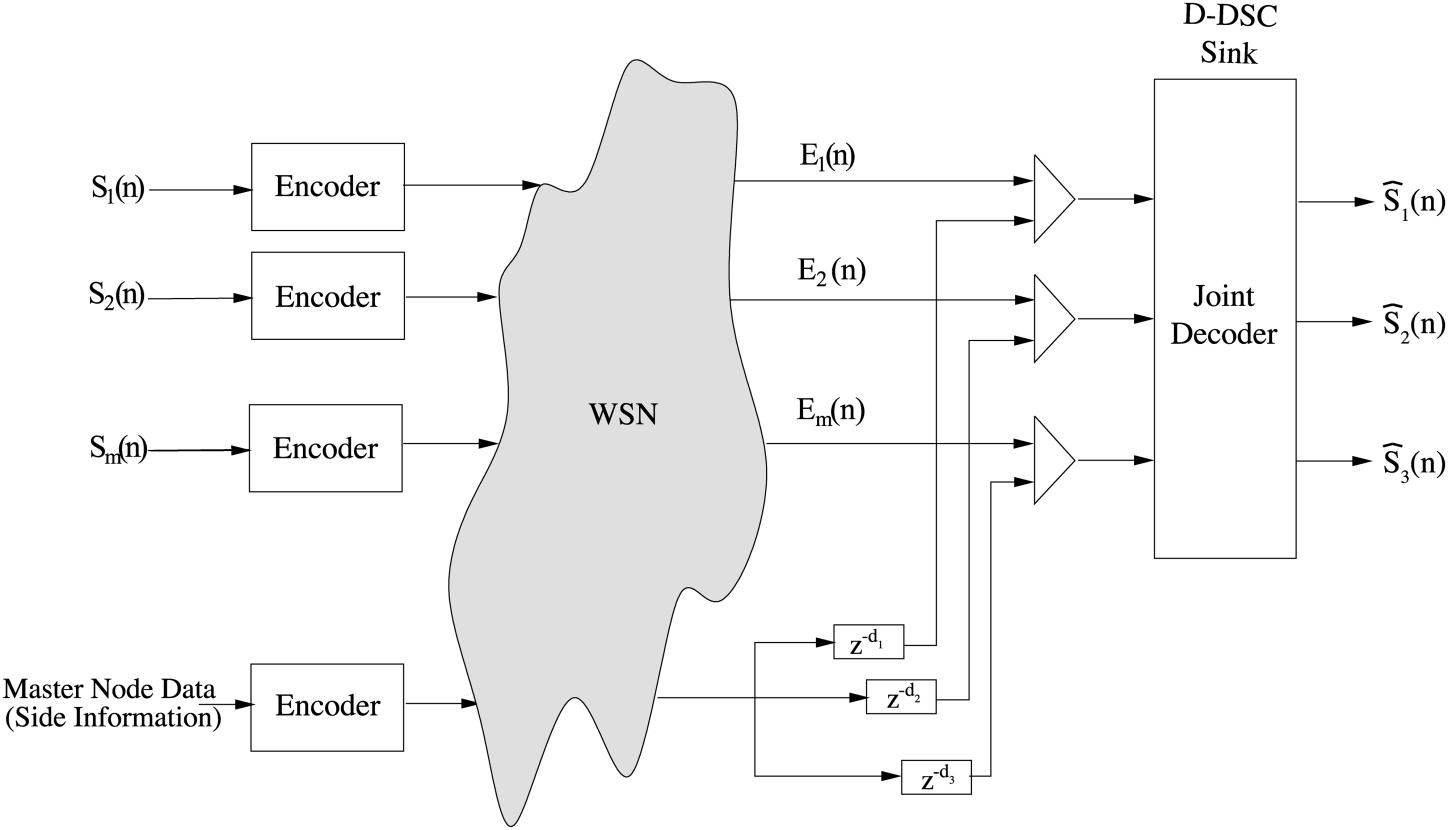
Joint decoding scheme of D-DSC.

The decoding scheme used for one cluster is illustrated in Fig 3. Here, the inputs for the joint decoder are the compressed data from slave nodes and uncompressed data from master node. The main difference of the joint decoder used in D-DSC compared to the classical DSC [10] is the use of different side information for each slave node, which is obtained by shifting the master node samples with different values. The amount of shift for the samples of each slave node is the *decoding delay*, *d*_*i*,*j*_, computed for each master-slave node pair (*i*, *j*) at the sink. Most importantly, it is possible to decode maximally compressed sensor node data with the same distortion bound (*D*_*max*_) achieved in classical DSC applications. Thus, for the same distortion bound, D-DSC promises for more power-efficient operation than classical DSC.

**Fig 4:**
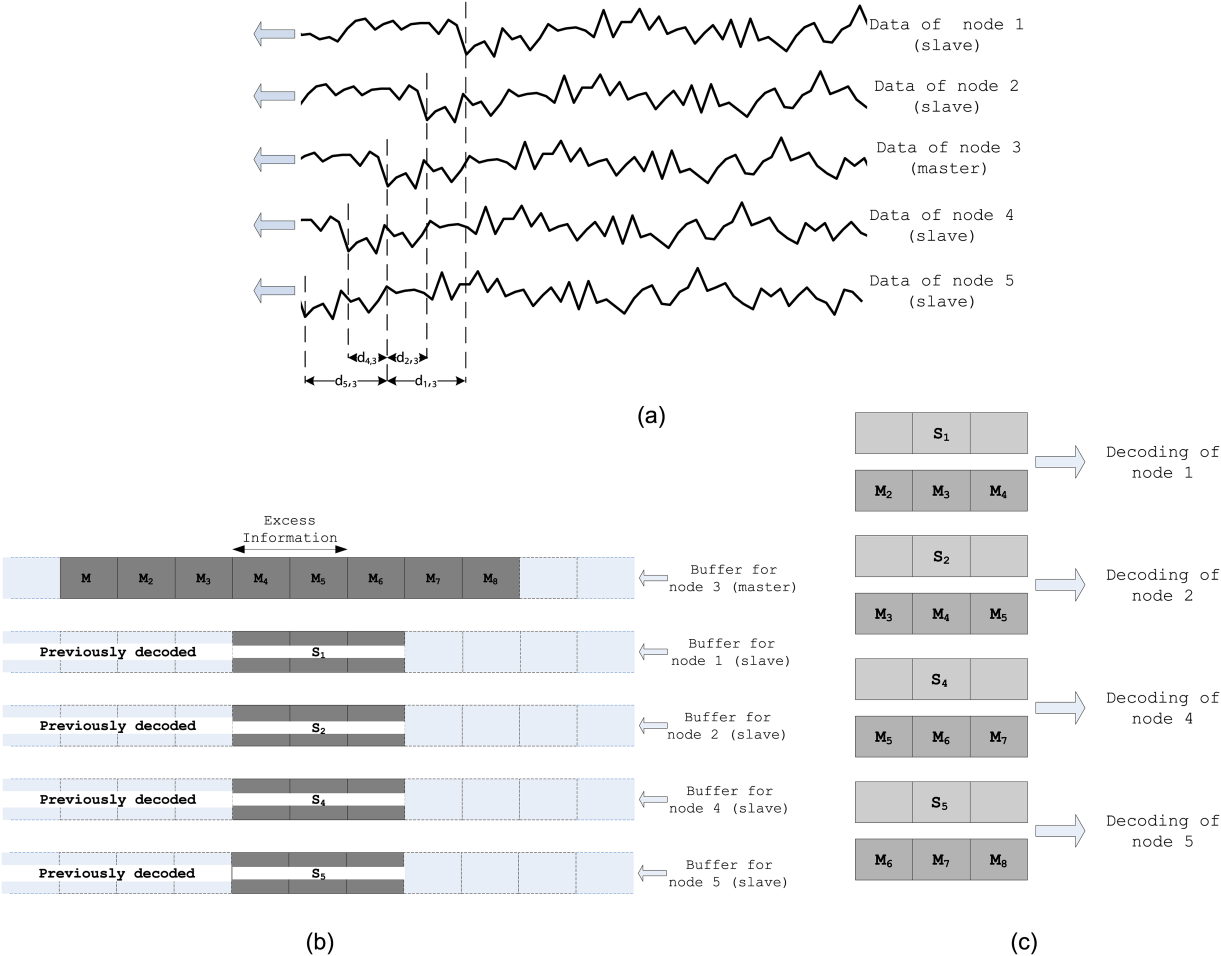
(a) Received raw data at sensor nodes based on the data model in (1) neglecting attenuation and noise, (b) Buffers on the joint decoder at the sink, (c) Decoding of slave nodes at the D-DSC sink.

The effectiveness of the proposed decoding scheme and the notion of decoding delay are elaborated with the following illustration. Let the five nodes are grouped into one cluster in which node 3 is selected as the master node. Based on the sensor data model in (1), samples of these nodes are illustrated in Fig 4a in which the propagation delays between sensor nodes are assumed to be, *d*_1,3_ = −2*d* *d*_2,3_ = −*d* *d*_4,3_ = *d* *d*_5,3_ = 2*d*. Here, the length of data packets in decision period (*P*_*d*_) and the excess information for master node are assumed to be *N* = 300 samples and *l*_*ex*_ = 200 samples, respectively. *d* corresponds to 100 data samples such as, *df*_*s*_ = 100. Then, the data at the buffer of joint decoder after the last packets are received is shown in Fig 4b, in which each block is assumed to contain 100 samples.

In D-DSC, the number of encoding bits *n*_*i*,*j*_ for each slave node is calculated by using the maximum correlation between master and slave node (10). Hence, to achieve desired distortion bound (*D*_*max*_), each slave node data should be decoded with the most similar data portion of the master node data. To realize this, the decoder should always contain the most similar data portion of master node with each slave node in the same cluster. In D-DSC, this condition is always guaranteed such as,

- *Slave node is closer to the event*—In this case, appropriate data portion is the advanced version of the master node data. Since at the end of the clustering process, the sink receives an excess information with the length of *l*_*ex*_ specified by (15) from only the master nodes, the decoder has always the master node data advanced with respect to the slave node data up to *l*_*ex*_ samples.
- *Master node is closer to the event*—In this case, appropriate data portion is the delayed version of the master node data. As initially all sensor nodes send an uncompressed data which are decoded separately, the decoder always has the master node data delayed with respect to the slave node data.

The decoding process of slave nodes’ data given in Fig 4(b) is illustrated in Fig 4(c).

#### 3.2.1 Network adaptation

Node failures, environmental factors and event mobility may change the network topology. D-DSC adapts the system parameters, *correlation function, decoding delays and cluster organization* to achieve the optimum compression rate within a given distortion bound (*D*_*max*_) in any topology.

D-DSC uses iterative-based correlation update in which only the current samples are used in correlation function estimation (9). In this method, it is also possible to control the effects of previous and current samples on the correlation function. The correlation update method used in D-DSC is defined by

$$\begin{array}{c} R_{i,j}^{U}\left(m\right)=\frac{\alpha R_{i,j}^{P}\left(m\right)+\beta R_{i,j}^{C}\left(m\right)}{\alpha +\beta }, \end{array}$$ (16)

where $R_{i,j}^{C}\left(m\right)$ is the current correlation estimate expressed by

$$\begin{array}{c} R_{i,j}^{C}\left(m\right)=\sum_{n=0}^{N-1}{\hat{x}}_{i}^{*}(n+PN-m){\hat{x}}_{j}(n+PN), \end{array}$$ (17)

and $R_{i,j}^{P}$ and $R_{i,j}^{U}$ are the previous and updated correlation estimates, respectively. ${\hat{x}}_{i}$ and ${\hat{x}}_{j}$ are the estimated sensor outputs obtained from joint decoder, and *P* is the number of previous decision periods. Coefficients *α* and *β* determine the effect of previous and current data samples on the correlation estimate, respectively. For fast topology changes, it is reasonable to select *α* < *β*, since the last packets provide more accurate information about the current state of the correlation in the network.

The correlation coefficients, *ρ*_*i*,*j*_, and decoding delays, *d*_*i*,*j*_, are recomputed by using the updated correlation functions, $R_{i,j}^{U}$, and then, used for determining the number of encoding bits (*n*_*i*,*j*_) for slave nodes.

A topology change may also affect the cluster organization. Therefore, the validity of the cluster should be periodically checked through a cluster verification procedure which is defined as follows. At the end of each decision period, the sink controls if the cluster conditions given in (12) are satisfied for each master-slave node pair. Any node that does not satisfy the conditions is taken out of the cluster. After controlling all of the clusters in the same way, the sink searches for a proper cluster for the nodes that do not belong to any cluster and joins them into this cluster if they satisfy the conditions in (12). If there still remain slave nodes that do not satisfy (12) for any given master node, the cluster organization described in Section 3.1 is performed again for these slave nodes. In this case, some slave nodes are converted to master node, and the sink requests each new master node for an extra uncompressed data with the length of *l*_*ex*_ as in the initialization phase. After the cluster update, the sink determines and broadcasts the number of encoding bits (10) for each sensor node again to resume D-DSC operation.

#### 3.2.2 Event estimation and reliability control

In D-DSC, the event is estimated based on the output of the joint decoder. The accuracy of the estimation directly depends on the packet delivery performance. Packets may not be delivered successfully to the sink for various reasons [3]. In classical WSN applications, due to highly redundant information on sensor data, the packet loss is not very effective on the event estimation performance, since the information in the lost packets can easily be compensated by the packets of other sensor data.

However, as D-DSC aims to minimize the redundancy in data transmission, many packets contain unique information about the event. Thus, packet losses may substantially degrade the estimation accuracy. Since the joint decoder uses the data of master node *j* as the side information and decodes the data of all the corresponding slave nodes with respect to the appropriate portion of the side information, 100% reliability is required for the packets generated by the master nodes. Otherwise, samples received from slave nodes in cluster *j* become useless in event estimation. This is not the case for slave nodes. Although the slave node samples are compressed with respect to master node samples in a maximum rate, there still exists spatial correlation among compressed sensor samples. Thus, slave nodes have relatively higher tolerance for packet loss.

For reliability, D-DSC determines the contribution of each sensor node on the event estimation and find the most required packets among the lost ones to achieve the desired event distortion bound, *D*_*max*_. Then these packets are retransmitted by using in-network caching algorithm which minimizes the number of retransmitted packets, and thus, the energy consumption during the reliability control.

Let the event *S* is estimated by linear estimation such as

$$\begin{array}{ccc} \hat{S}\left(n\right) & = & \frac{1}{M}\sum_{i=1}^{M}\gamma_{i}{\hat{x}}_{i}\left(n+\tau_{i}\right),\;n=1,2,\ldots ,L\\ \hat{\underline{S}} & = & \frac{1}{M}\underline{\Gamma }X, \end{array}$$ (18)

where *τ*_*i*_ is the propagation delay between the event source and sensor node *i*, and

$$\begin{array}{ccc} \underline{\Gamma } & = & \left[\gamma_{1}\;\gamma_{2}\;\ldots \;\gamma_{M}\right],\\ X & = & {\left[{\underline{\hat{x}}}_{1}\;{\underline{\hat{x}}}_{2}\;\ldots \;{\underline{\hat{x}}}_{M}\right]}^{T}, \end{array}$$ (19)

where ${\underline{\hat{x}}}_{i}={\left[{\hat{x}}_{i}\left(1+\tau_{i}\right)\ldots {\hat{x}}_{i}\left(L+\tau_{i}\right)\right]}^{T}$ is the estimated sensor node data obtained at the joint decoder and shifted by *τ*_*i*_; *γ*_*i*_ is the weighting coefficient which is a function of decoding delay between sensor samples and attenuation coefficient, *λ*, in (1). *M* and *L* are the number of sensors and samples, respectively.

In a similar way, the event estimation obtained using *k* received packets at the sink can be defined as

$$\begin{array}{ccc} {\hat{S}}_{k}^{r}\left(n\right) & = & \frac{1}{k}\sum_{i\in \{{}_{packets}^{received}\}}\gamma_{i}{\hat{x}}_{i}\left(n+\tau_{i}\right),\;n=1,2,\ldots ,L\\ {\hat{\underline{S}}}_{k}^{r} & = & \frac{1}{k}{\underline{\Gamma }}_{k}^{r}X_{k}^{r}, \end{array}$$ (20)

where the superscript *r* is used for denoting *received packets* and subscript *k* is the number of received packets. Then, the event distortion for *k* packets can be defined as

$$D_{e}^{k}=\frac{E\left\{{\left\|\hat{\underline{S}}-{\hat{\underline{S}}}_{k}^{r}\right\|}^{2}\right\}}{E\left\{{\left\|\hat{\underline{S}}\right\|}^{2}\right\}},$$ (21)

Assume that the sink receives one more packet labeled as ${\underline{\hat{x}}}_{p}$ to use in the event estimation. Then, the event distortion for *k* + 1 packets is found as,

$$\begin{array}{c} D_{e}^{k+1}=\frac{E\left\{{\left\|\hat{\underline{S}}-{\hat{\underline{S}}}_{k+1}^{r}\right\|}^{2}\right\}}{E\left\{{\left\|\hat{\underline{S}}\right\|}^{2}\right\}}. \end{array}$$ (22)

Using (20), ${\hat{\underline{S}}}_{k+1}^{r}$ can be written in terms of ${\hat{\underline{S}}}_{k}^{r}$, i.e.,

$$\begin{array}{ccc} {\hat{\underline{S}}}_{k+1}^{r} & = & \frac{1}{k+1}{\underline{\Gamma }}_{k+1}^{r}X_{k+1}^{r}\\ & = & \frac{1}{k}{\underline{\Gamma }}_{k}^{r}X_{k}^{r}-\frac{1}{k(k+1)}{\underline{\Gamma }}_{k}^{r}X_{k}^{r}+\frac{1}{k+1}\gamma_{p}{\underline{\hat{x}}}_{p},\\ & = & {\hat{\underline{S}}}_{k}^{r}+{\underline{\Psi }}_{k}. \end{array}$$ (23)

Substituting (23) into (22), we obtain the relation between $D_{e}^{k+1}$ and $D_{e}^{k}$, i.e.,

$$\begin{array}{ccc} D_{e}^{k+1} & = & \frac{E\left\{{\left\|\hat{\underline{S}}-{\hat{\underline{S}}}_{k}^{r}-{\underline{\Psi }}_{k}\right\|}^{2}\right\}}{E\left\{{\left\|\hat{\underline{S}}\right\|}^{2}\right\}}=\frac{E\left\{{\left\|\hat{\underline{S}}-{\hat{\underline{S}}}_{k}^{r}\right\|}^{2}\right\}}{E\left\{{\left\|\hat{\underline{S}}\right\|}^{2}\right\}}-\frac{E\left\{2ℜ\left\{\left(\hat{\underline{S}}-{\hat{\underline{S}}}_{k}^{r}\right){\left({\underline{\Psi }}_{k}\right)}^{H}\right\}-{\left\|{\underline{\Psi }}_{k}\right\|}^{2}\right\}}{E\left\{{\left\|\hat{\underline{S}}\right\|}^{2}\right\}},\\ & = & D_{e}^{k}-D_{p}^{l} \end{array}$$ (24)

where, $D_{p}^{l}$ is the degradation in the event distortion when samples of node *p* is used in event estimation, and superscript *l* denotes the *lost packets*. Among all these lost packets, the most informative for the event estimation is the one that yields the maximum degradation in the event distortion $D_{e}^{k}$, and determined as

$$\begin{array}{cc} p^{*}= & argmax\{D_{p}^{l}\},\\ & p\in \{{}_{packets}^{lost}\}. \end{array}$$ (25)

The second most informative packet can be found using (25) for the updated set of *lost packets* (excluding node p*). Repeating this procedure for the entire set generates an importance sequence for event estimation such as

$$\begin{array}{c} D_{p_{1}^{*}}>D_{p_{2}^{*}}>\ldots >D_{p_{M_{l}}^{*}}, \end{array}$$ (26)

where $D_{p_{i}^{*}}$ is the degradation in the event distortion when samples of node $p_{i}^{*}$ is received at the *i*^*th*^ retransmission and used in the event estimation, and *M*_*l*_ is the number of lost packets. Then, the minimum number of sensor nodes for which retransmission is requested can be determined as

$$p\in \{\begin{array}{cc} \left\{p_{1}^{*},\ldots ,p_{s}^{*}\right\}s.t. & \begin{array}{c} D_{e}^{k}-\sum_{i=1}^{s}D_{p_{i}^{*}}\leq D_{max}<D_{e}^{k}-\sum_{i=1}^{s-1}D_{p_{i}^{*}}\\ D_{max}<D_{e}^{k} \end{array},\\ \left\{\emptyset \right\}, & D_{max}\geq D_{e}^{k}, \end{array}$$ (27)

where *D*_*max*_ is the maximum event distortion allowed by the application, and $\sum_{i=1}^{s\leq 0}D_{p_{i}^{*}}=0$. The notation {⌀} denotes that no retransmission is required.

**Algorithm 1** Pseudocode for the Sink Operation

Given *S*_*U*_: set of undefined sensors; *S*_*M*_: set of master nodes and corresponding clusters; *S*_*S*,*j*_: set of slaves in cluster *j*; *P*: period number of decision; *N*: number of samples received in a decision period; *I*: number of samples requested in initialization process

1: **procedure** Main

2:  **for** (*k* = 0; *k* < *I*; *k*++) **do**

3:   **for** (*i* = 0; *i* < *M*; *i*++) **do**

4:    request node *i* for uncoded reading of *k*th sample

5:   **end for**

6:  **end for**

7:  **for all** node pairs (*i*, *j*) **do**

8:   calculate correlation parameters ***ρ*** and ***d*** using (11) and (8)

9:  **end for**

10:  *Cluster*(*S*_*U*_) in Algorithm 2

11:  **for** (*P* = 1; *P*++) **do**

12:   determine *n*_*ij*_ for each slave-master pairs (*i*, *j*) using (10)

13:   **for** (*k* = *I* + (*P* − 1)*N*; *k* < *I* + *PN*; *k*++) **do**

14:    **for all** *j* ∈ *S*_*M*_ **do**

15:     request node *j* for uncoded reading of (*k* + *l*_*ex*_)th sample

16:     **for all** *i* ∈ *S*_*S*,*j*_ **do**

17:      request node *i* for *n*_*ij*_-bit reading of *k*th sample

18:     **end for**

19:    **end for**

20:   **end for**

21:   **if** there are lost packets **then**

22:    request for retransmissions based on (25)–(27)

23:   **end if**

24:   decode all bits received in period *P* as explained in Section 3.2

25:   estimate event for period *P* using (20)

26:   update correlations ***R*** using (16)

27:   *ClusterValid*(***ρ***, ***d***) in Algorithm 2

28:  **end for**

29: **end procedure**

**Algorithm 2** Pseudocode for Cluster Formation and Validation Functions

Given *S*_*U*_: set of undefined sensors; *S*_*M*_: set of master nodes and corresponding clusters; *S*_*S*,*j*_: set of slaves in cluster *j*; *P*: period number of decision; *N*: number of samples received in a decision period; *I*: number of samples requested in initialization process

1: **function** Cluster(*S*_*U*_)

2:  **while** *S_U_* ≠ ⌀ **do**

3:   find node *j* in *S*_*U*_ with largest set of correlated nodes in *S*_*U*_\*j* satisfying clustering conditions (12)

4:   *S*_*M*_ ← *S*_*M*_ ∪ *j*; create *S*_*S*,*j*_

5:   *S*_*U*_ ← *S*_*U*_\(*j* ∪ *S*_*S*,*j*_)

6:  **end while**

7:  **for** (*s* = *k*; *s* < *s* + *l*_*ex*_; *s*++) **do**

8:   **for all** *j* ∈ *S*_*M*_ **do**

9:    ask node *j* for uncoded reading of *s*th sample

10:   **end for**

11:  **end for**

12: **end function**

13: **function** ClusterValid(***ρ***, ***d***)

14:  **for all** node pairs (*j* ∈ *S*_*M*_, *i* ∈ *S*_*S*,*j*_) **do**

15:   **if** *ρ*_*ij*_ > *ρ*_*th*_ **OR** *d*_*ij*_ > *d*_*th*_ **then**

16:    *S*_*S*,*j*_ ← *S*_*S*,*j*_\*i*

17:    **if** *ρ*_*ig*_ < *ρ*_*th*_ **AND** *d*_*ig*_ < *d*_*th*_ for any node *g* ∈ *S*_*M*_\*j* **then**

18:     determine *g* with largest *ρ*_*ig*_; *S*_*S*,*g*_ ← *S*_*S*,*g*_ ∪ *i*

29:    **else**

20:     *S*_*U*_ ← *S*_*U*_ ∪ *i*

21:    **end if**

22:   **end if**

23:  **end for**

24:  **if** *S_U_* ≠ ⌀ **then**

25:   *Cluster*(*S*_*U*_)

26:  **end if**

27: **end function**

To minimize the number of retransmissions, in-network caching is performed. Each sensor node has an internal cache to store its data as well as the data received from other sensor nodes during multi-hop transmission. The maximum memory required for the internal cache is, *MNn*_*u*_, where *N* is the number of samples in a decision period (*P*_*d*_). Whenever a retransmission is requested for node *i*, the required packet is provided from the internal cache of a node nearest to the sink instead of the node *i*, which may be far away from the sink.

Once the sink determines the lost packets required for achieving the desired event distortion (27), it broadcasts the *selective retransmission request* (SRR) packet which contains the IDs of the requested lost packets. During multi-hop transmission, each sensor node receiving the SRR packet checks in its cache whether any of the packets matches with the IDs in the SRR packet. If it does not have any match, it forwards the SRR packet to the next sensor node in the routing path without any change. If the match is found, the matching IDs are removed from the SRR packet, and the lost packets with these IDs are sent to the sink. Then, if there is remaining IDs in the modified SRR packet, it is forwarded again. This procedure is repeated until there remains no ID in the SRR packet. Therefore, the number of transmissions for each lost packet is minimized and it results more energy savings.

Note that D-DSC requires the propagation delay between the source and sensor nodes for the event estimation as in (18). In fact, many existing methods for source and sensor localization [31] may be used to estimate the propagation delays, and its details are beyond the scope of our work.

## 4 Performance evaluation

We model an event monitoring application, in which real data records are used as an event. Three types of data are used in the simulations: *music record in wav format, randomly generated signal at 500*Hz* and a real temperature measurement record*. Simulation environment is created using MATLAB. Sensor nodes, sink and event source are randomly located on the event area and the data model in (1) is utilized. The simulation parameters are given in Table 1.

**Table 1:** System parameters used in simulations.

Parameter	Music	Random	Temperature
*# of sensors*	15	15	15
*Deployment area*	50m×50m	50m×50m	100m×100m
*Correlation thresh*. (*ρ*_*th*_)	0.95	0.95	0.95
*Delay thresh*. (*d*_*th*_)	0.5s	0.5s	1000s
*Samples in decision* (*N*)	750	200	25
*# of uncoded bits* (*n*_*u*_)	8bits	8bits	8bits
*Packet loss rate* (*L*_*p*_)	0	0	0
*Sampling frequency* (*f*_*s*_)	8kHz	2kHz	0.002Hz
*Propagation speed* (*v*_*s*_)	330m/s	330m/s	2.5m/s
*Attenuation coeff*. (*λ*)	0.0048	0.0048	0.001
*Signal to noise ratio*	30dB	30dB	50dB

Since the power consumption in WSNs is dominated by communication, compression rate performance becomes significant for WSN applications in terms of energy efficiency. We define the compression rate as the ratio between the total number of bits used in communication for D-DSC and the application that does not use any source coding technique. In addition, event distortion is measured as mean-square error between the source data and the event estimation at the sink.

### 4.1 Clustering thresholds

**Fig 5:**
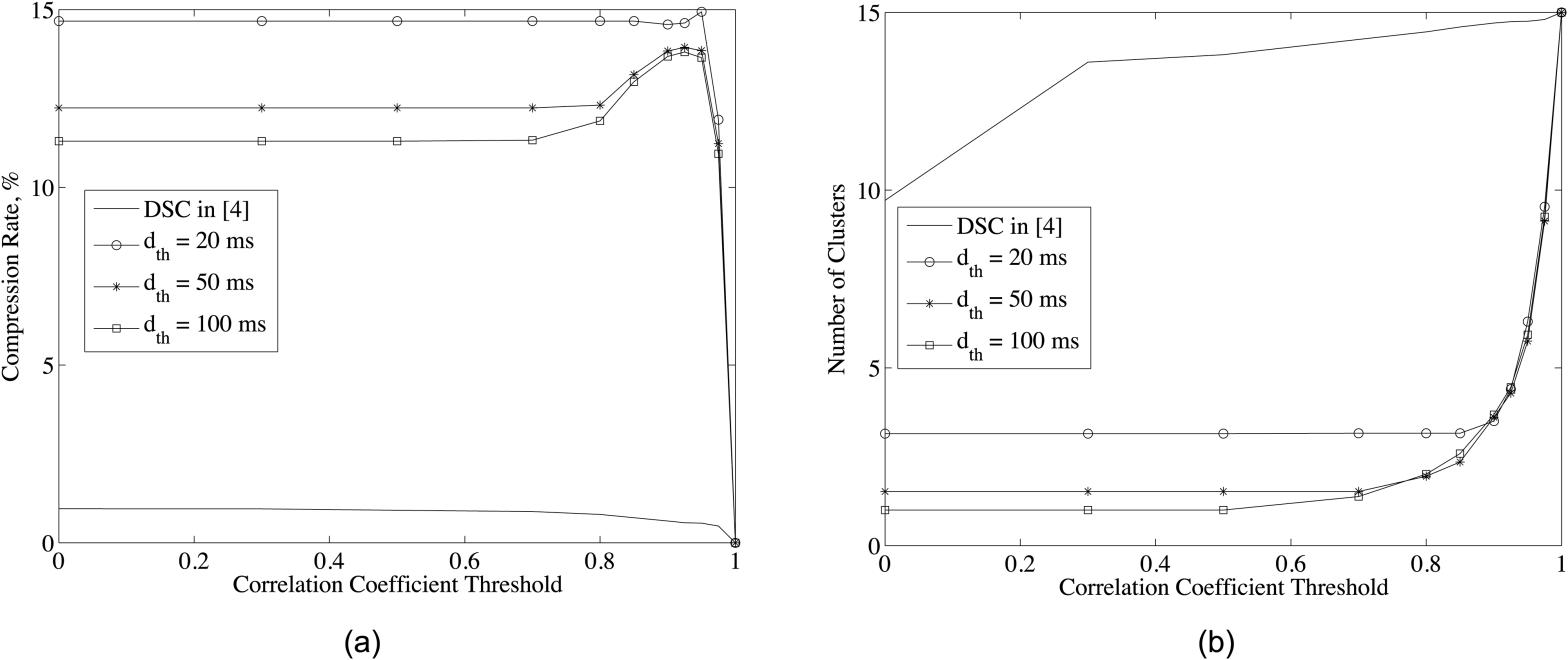
(a) Compression rate and (b) the number of clusters for different correlation thresholds and delay thresholds and system parameters given in Table 1.

D-DSC is a cluster-based solution and clusters are determined jointly by correlation coefficient threshold (*ρ*_*th*_) and delay threshold (*d*_*th*_) in (12). Therefore, we evaluate the compression rate metric and the number of clusters with respect to *ρ*_*th*_ for different *d*_*th*_ values for music record. Zero delay threshold case is considered as no decoding delay concept as in the classical DSC [10]. Increasing correlation coefficient threshold has two effects on the performance, namely increasing the compression rate in each cluster and increasing the number of clusters. While the first one decreases the power consumption, the second one increases it because in each cluster master node sends its uncompressed samples. Thus, the trade-off between the number of clusters and compression rate in each cluster which results optimum *ρ*_*th*_ (14) should be considered for minimum power consumption. As in Fig 5a, the optimum *ρ*_*th*_ is approximately 0.95. As the delay threshold decreases, the effect of *ρ*_*th*_ on cluster selection is restricted and clusters are mostly determined by *d*_*th*_ as in (12) and shown in Fig 5b. Similar result is valid for large *ρ*_*th*_ case in which clusters are mostly determined by *ρ*_*th*_. Hence, for the time-critical applications, there is no need to search for optimum *ρ*_*th*_, setting it as 0 suffices for minimum power consumption. However, for the other applications, optimum *ρ*_*th*_ should be determined as in (12) for minimum power consumption.

D-DSC method provides significant gains in the data compression at the expense of additional delay in the network. Therefore, the proposed algorithm is especially promising for delay tolerant IoST applications, in which the delay thresholds are not strict. Smart city applications can be considered as a promising venue for D-DSC algorithm. Smart city concept includes the realization of various WSN in the city to monitor and optimize the physical infrastructure. To this end, some of WSN applications, e.g. temperature sensors to detect the medium to long term climate changes, can utilize D-DSC concept having a few 100s to 1000 second delay thresholds [32, 33]. Since IoST includes various sources of data, D-DSC is promising to enhance efficiency of the networks for data sources, which may favor higher compression rates at the expense of additional delay.

The benefits of the proposed decoding delay concept can be better observed with respect to zero delay threshold case [10]. As shown in Fig 5a, the decoding delay concept can provide 10 times better compression rate than the DSC [10].

### 4.2 Decision period

**Fig 6:**
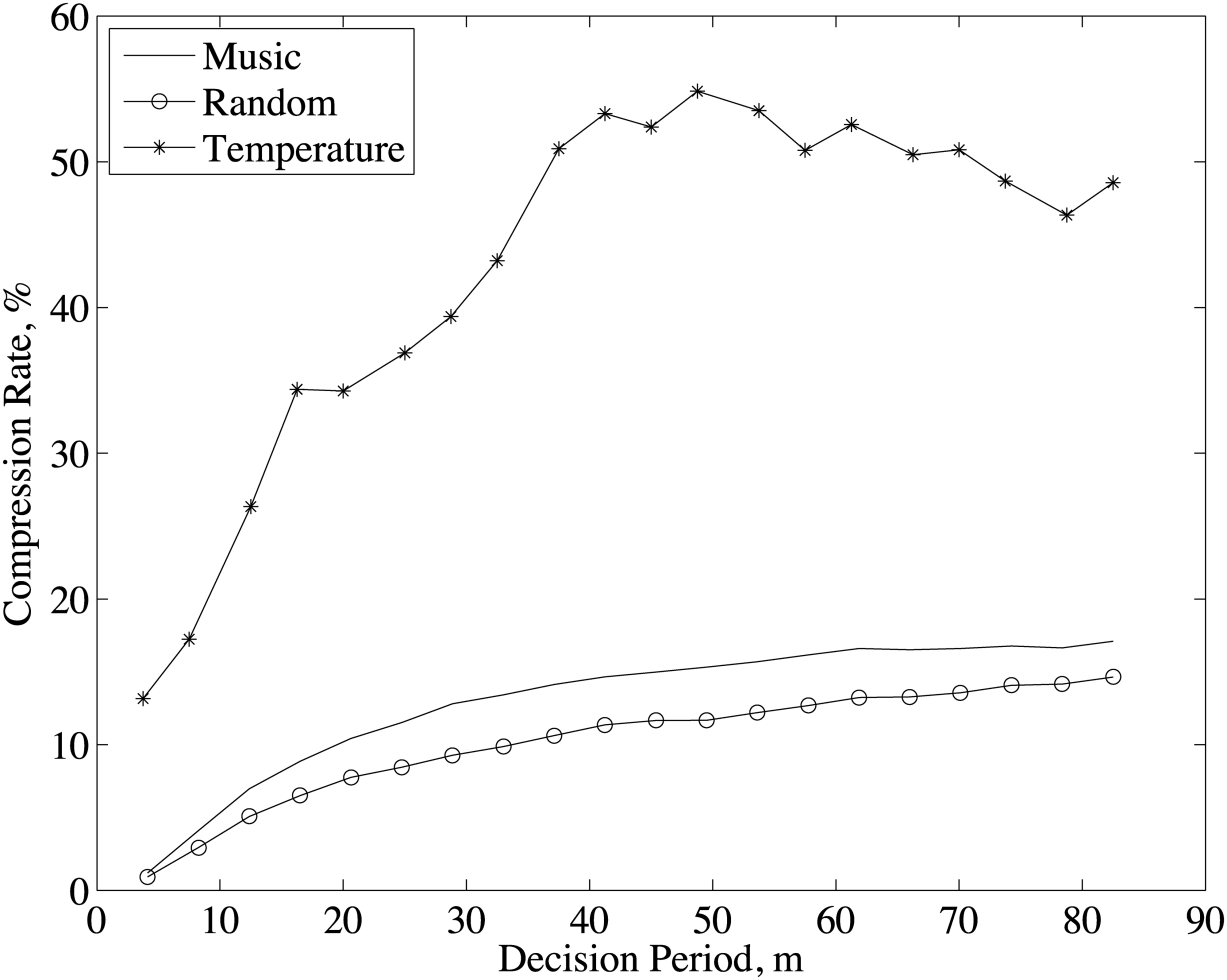
Compression rate for different decision period and system parameters given in Table 1.

Decision period (*P*_*d*_) is the spatial period which defines the maximum distance between two sensor nodes whose samples can be maximally correlated at the joint decoder, i.e., $P_{d}=\frac{N}{f_{s}}v_{s}$. The effect of *P*_*d*_ on compression rate is shown in Fig 6 for three data types. Since the sampling frequency of temperature data is very low (*f*_*s*_ = 0.002 *Hz*), the decision period for this data type is in *kilometer* while for the others it is in *meter*. As noticed in Fig 6, increasing decision period increases the compression rate (ϒ) for all data types and compression rate of temperature data is much higher than the other data types. As in Section 3.2, D-DSC achieves the maximum compression by using the most correlated data portion of sensor samples, which is determined by the correlation lags. Therefore, *the largest correlation lag among the clusters should be within the data size in P_d_ for the achievable maximum compression rate*. Increasing *P*_*d*_ up to the largest correlation lag increases the compression rate. On the other hand, it also decreases the adaptation capability for topology changes because the network is reorganized after processing the data in *P*_*d*_. Note that the achievable maximum compression rate also depends on the selection of *ρ*_*th*_ and *d*_*th*_. For the optimum *ρ*_*th*_, the minimum data size in *P*_*d*_ that gives the maximum compression is limited with $\min\{\left(f_{s}d_{th}\right),\max_{i,j}\left\{d_{i,j}\right\}\}$, where *f*_*s*_ is the sampling frequency and *d*_*i*,*j*_ is the decoding delay between sensor node *i* and *j*.

### 4.3 Signal to noise ratio

**Fig 7:**
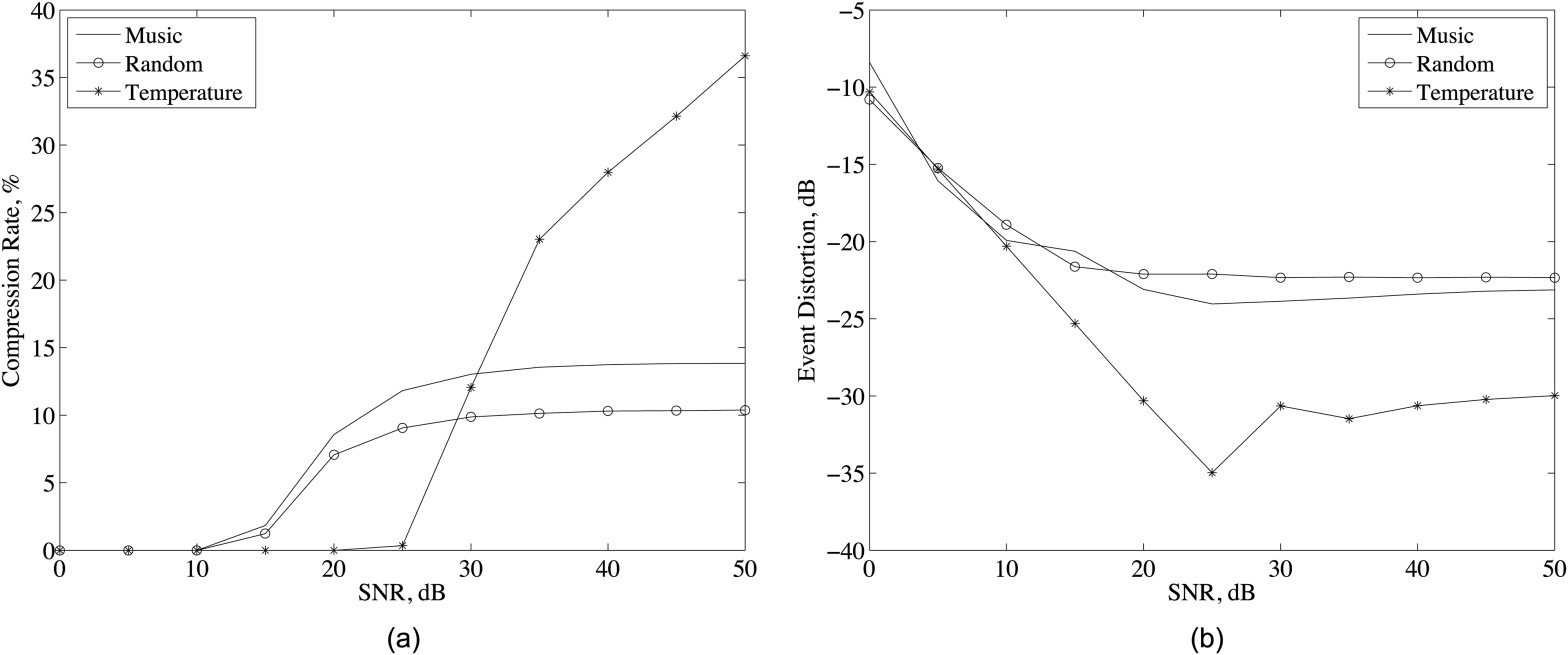
(a) Compression rate and (b) event distortion for different SNR values and system parameters given in Table 1.

Signal-to-noise-ratio (SNR) of an event signal affects the compression rate performance as in Fig 7a. We assume that the noise is additive white noise and SNR is defined as the ratio of event signal power to the noise power. As noticed, increasing SNR increases the compression rate for all data types. Since the noise is uncorrelated for all sensors, the measured sensor data becomes less correlated as the effects of the noise increases with the decreasing SNR values. As in Fig 7a, while the compression rate of music record and randomly generated signal is flattened after 30 dB SNR, the compression rate continues to increase for the temperature data. In addition, the compression rate of temperature data is less than the music record and randomly generated signal for small SNR values because the temperature data is much smoother than the other data types and even for the large SNR values. On the other hand, for the small SNR values, the correlation between the data of sensor nodes is more sensitive to noise for temperature data. The result of these observations can be better understood from signal frequency analysis given in the next subsection.

The effect of SNR on event distortion is shown in Fig 7b for three data types. Since D-DSC reconstructs the event signal from samples of the sensor nodes (18), the noise in the event signal directly affects the accuracy of the samples and event estimation. Increasing SNR decreases the noise power and hence, the event distortion as in Fig 7b. However, further increasing in SNR does not decrease the event distortion. because the noise power is small at high SNR values and, the major effect on the sample accuracy becomes the decoding error, which does not depends on SNR. As noticed in Fig 7b, event distortion of temperature data is much lower than the other data types. Thus, we can state that the decoding error becomes less for smoother signals as in the temperature case.

### 4.4 Angular frequency of signal

**Fig 8:**
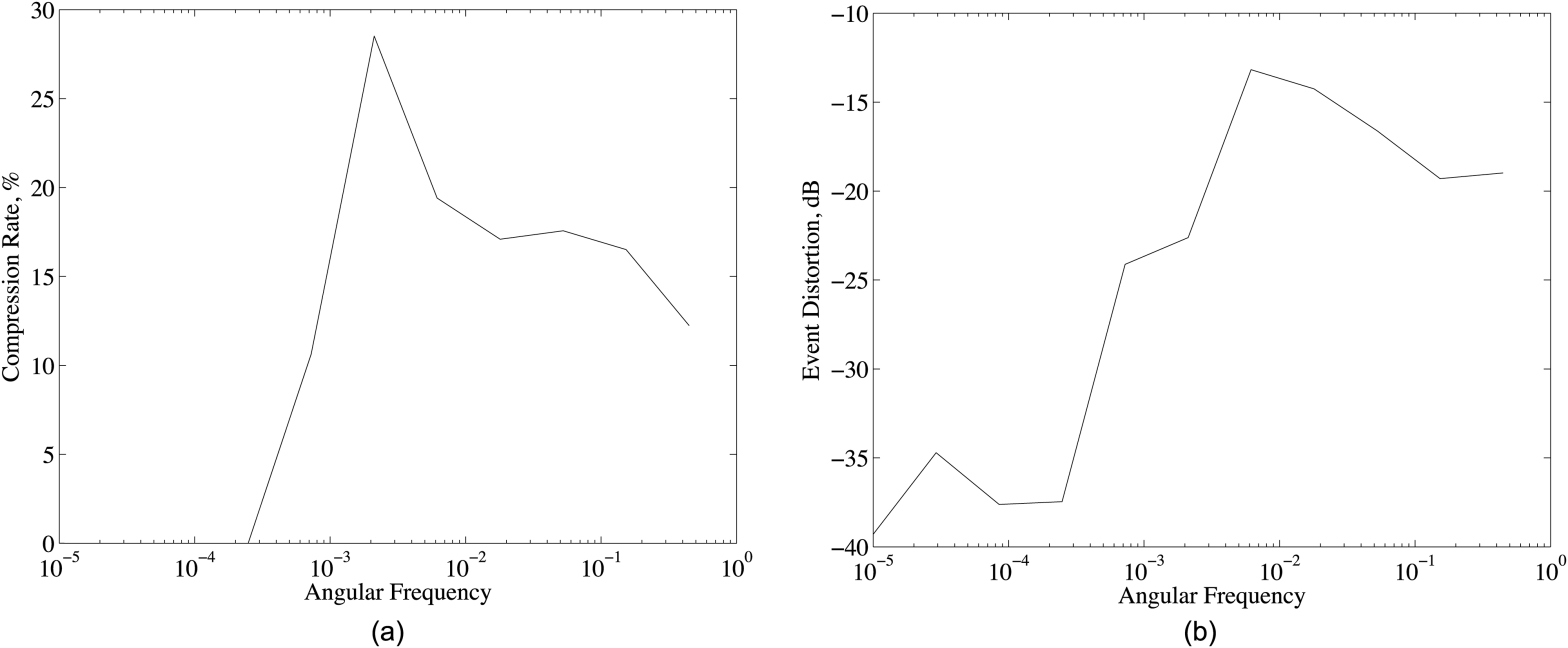
(a) Compression rate and (b) event distortion for different angular frequency of the source and system parameters given in Table 1.

Angular frequency (*F*_*sig*_) is the frequency of the event signal normalized by the half of the sampling frequency, and it determines the smoothness of the sensor samples. Its effects on compression rate performance are evaluated by using the low-pass filtered version of the randomly generated signal as an event. As in Fig 8a, increasing angular frequency at first increases the compression rate and then decreases it. Lower *F*_*sig*_ results in smoother sensor samples for the same data size in packets, and hence, larger degree of correlation between sensor samples. However, as in Fig 8a, at low angular frequencies, the sensor samples in *P*_*d*_ are almost constant and even for the large SNR values noise signal can significantly reduce the correlation between sensor samples. As *F*_*sig*_ increases, the effects of noise on correlation decreases and compression rate increases. Further increasing *F*_*sig*_ again decreases the correlation between sensor samples, however in this case, the reason is not the noise signal but the decreasing smoothness in sensor samples. Thus, the angular frequency is an important parameter for compression rate performance of D-DSC.

Angular frequency also affects the event distortion. As in Fig 8b, the event distortion increases with angular frequency. D-DSC reconstructs the event signal from the sensor samples and the propagation delay between event and sensor nodes by using linear estimation given in (18). Here, we assume that the propagation delay between event and sensor samples are exactly known, and hence, Fig 8b shows only the angular frequency effect on event distortion. If the time difference between propagation delay of two sensor nodes is not the integer multiples of the sampling period, sensor nodes take samples from the event signal at different points in time which reduces the correlation between samples of sensor nodes. This case is highly probable for randomly deployed sensor nodes. Since increasing angular frequency reduces the smoothness of the observed signal, the correlation between sensor samples reduces with increasing angular frequency. Thus, the angular frequency should be as low as possible for the minimum event distortion. However, this is not the case for compression rate as in Fig 8a, which yields an important trade-off that should be considered.

### 4.5 Node density and number of nodes

**Fig 9:**
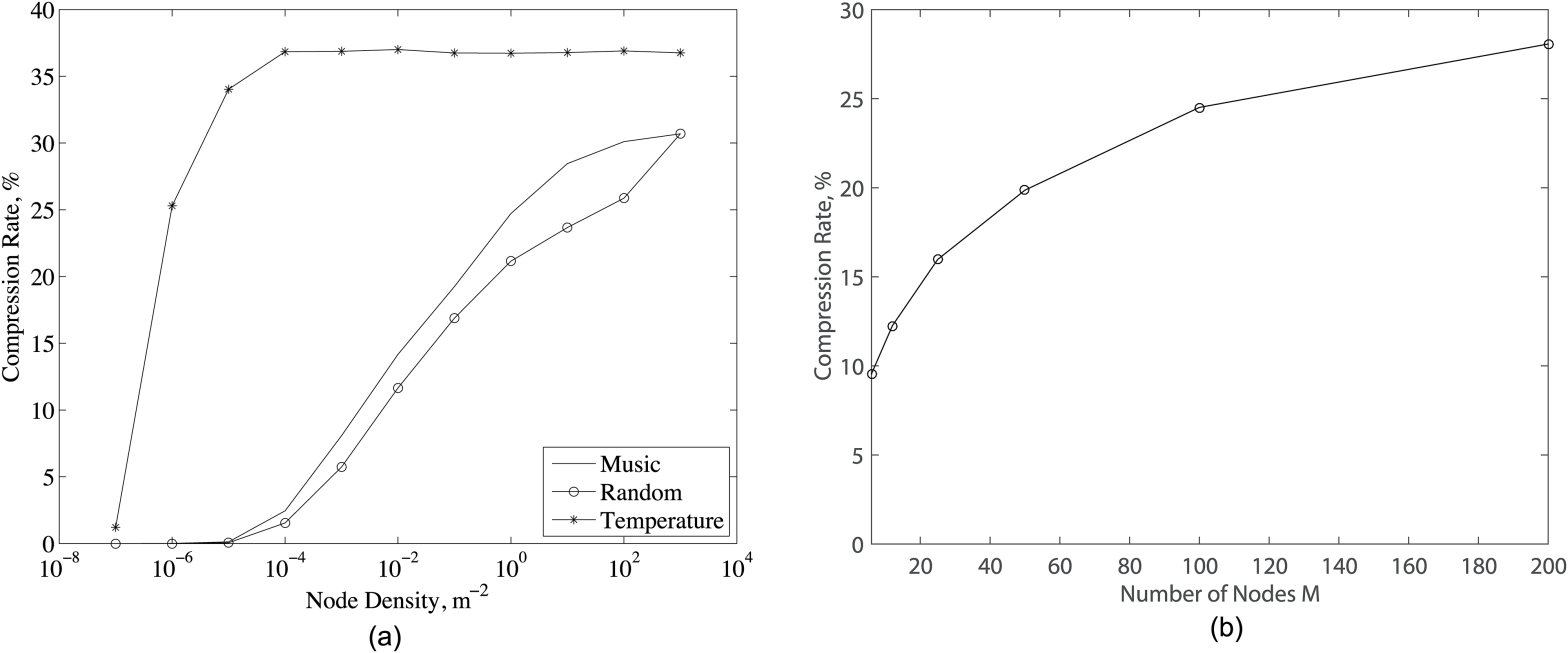
(a) Compression rate for different node density and system parameters given in Table 1. (b) Compression rate of music data sample of *N* = 100000 bits for different number of nodes *M* and system parameters given in Table 1 with deployment area being set to 25*m* × 25*m* for *M* = 6, $25\sqrt{2}m\times 25\sqrt{2}m$ for *M* = 12, 50*m* × 50*m* for *M* = 25, $50\sqrt{2}m\times 50\sqrt{2}m$ for *M* = 50, 100*m* × 100*m* for *M* = 100, $100\sqrt{2}m\times 100\sqrt{2}m$ for *M* = 200 to have a stationary node density.

Fig 9a presents the compression rate vs. node density for all data types. As noticed, compression rate increases with node density because the correlation between the sensor data increases in more closely placed sensors. As explained in Section 4.2, D-DSC achieves the maximum compression rate when maximum distance between sensor nodes is smaller than *P*_*d*_, which is the case for temperature data after node density of 10^−4^. Therefore, the compression rate of temperature data is approximately the same for the node density of 10^−4^ m^−2^ as in Fig 9a. Since this condition is not the case for audio and randomly generated signal, their compression rates continue to increase with increasing node density. Fig 9b presents the results for the compression rates for fixed node density for music data sample. As noticed, the compression rate improves as the number of data sources are increasing since the likelihood of correlation between sources becomes higher with the increasing number of data source. However, the rate of increase in the compression rate reduces and saturates between 25–30%. This effect is caused by the decreasing correlation between sensor data due to increasing distance between sensor nodes due to constant node density.

### 4.6 Network adaptation

For network adaptation results, 15 nodes are randomly deployed on the area of [160000 m ×100 m] and a heat source initially located at (0, 0) is moved with constant velocity over on the x-axis. The maximum velocity of the source is selected as the 10% of the propagation speed of the event signal (*v*_*s*_ = 2.5 m/s). 10% of nodes are determined as malfunctioned at a randomly selected time instant and 5% of packets are lost during transmission. In addition to these, the nodes are determined as dead nodes after transmitting more than *E*_*node*_ = 12000 bits and not used any more. Since the cluster verification algorithm in D-DSC continuously updates the cluster organization such that the most appropriate nodes are used for event monitoring, the cluster verification is expected to improve both the compression rate and event distortion performance of the network.

**Fig 10:**
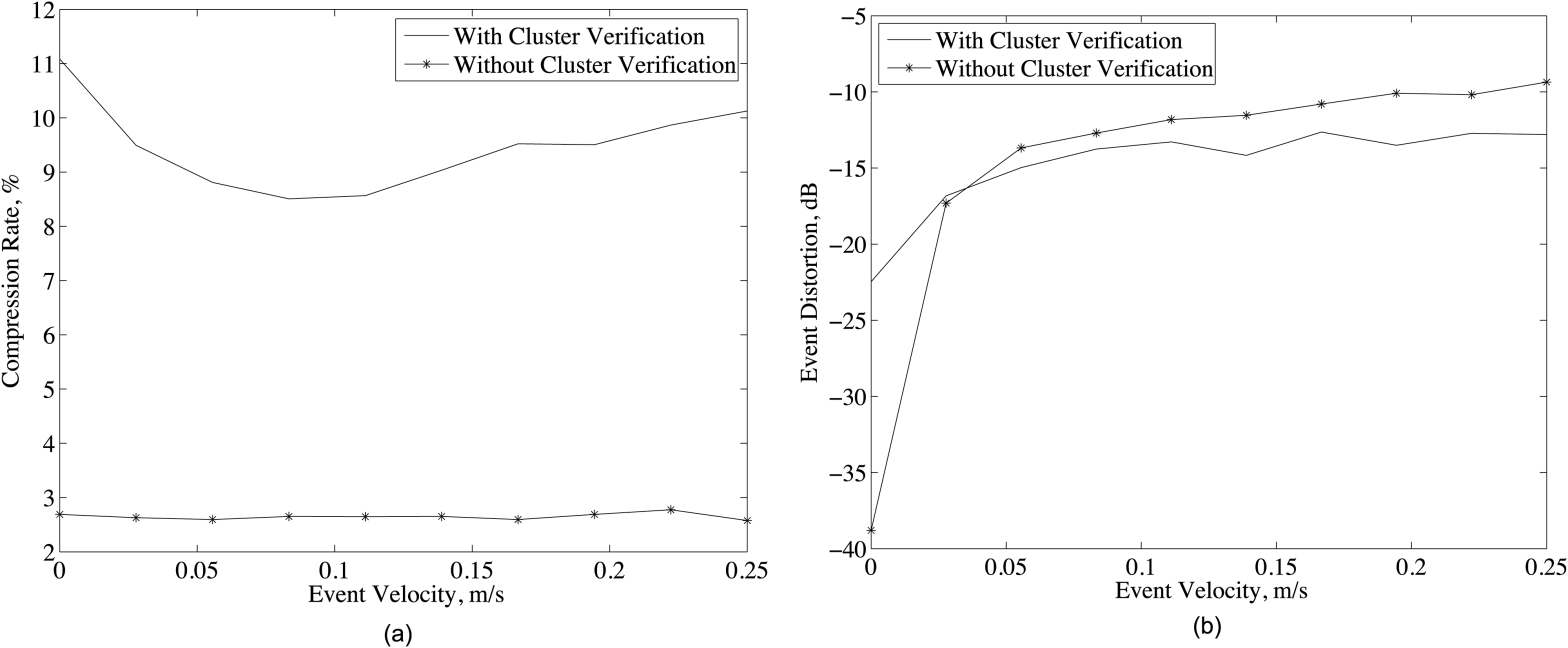
(a) Compression rate and (b) event distortion for different event velocity and system parameters given in Table 1 with *L*_*p*_ = 0.05, *R*_*mal*_ = 10%, *E*_*node*_ = 12000 bits.

As shown in Fig 10a, when cluster verification is applied, the compression rate performance of D-DSC significantly improves compared to the case that cluster verification is not applied. In addition, the even distortion is significantly reduced with the cluster verification as in Fig 10b. Thus, D-DSC becomes robust to highly varying network dynamics with the cluster verification.

### 4.7 Packet loss rate

**Fig 11:**
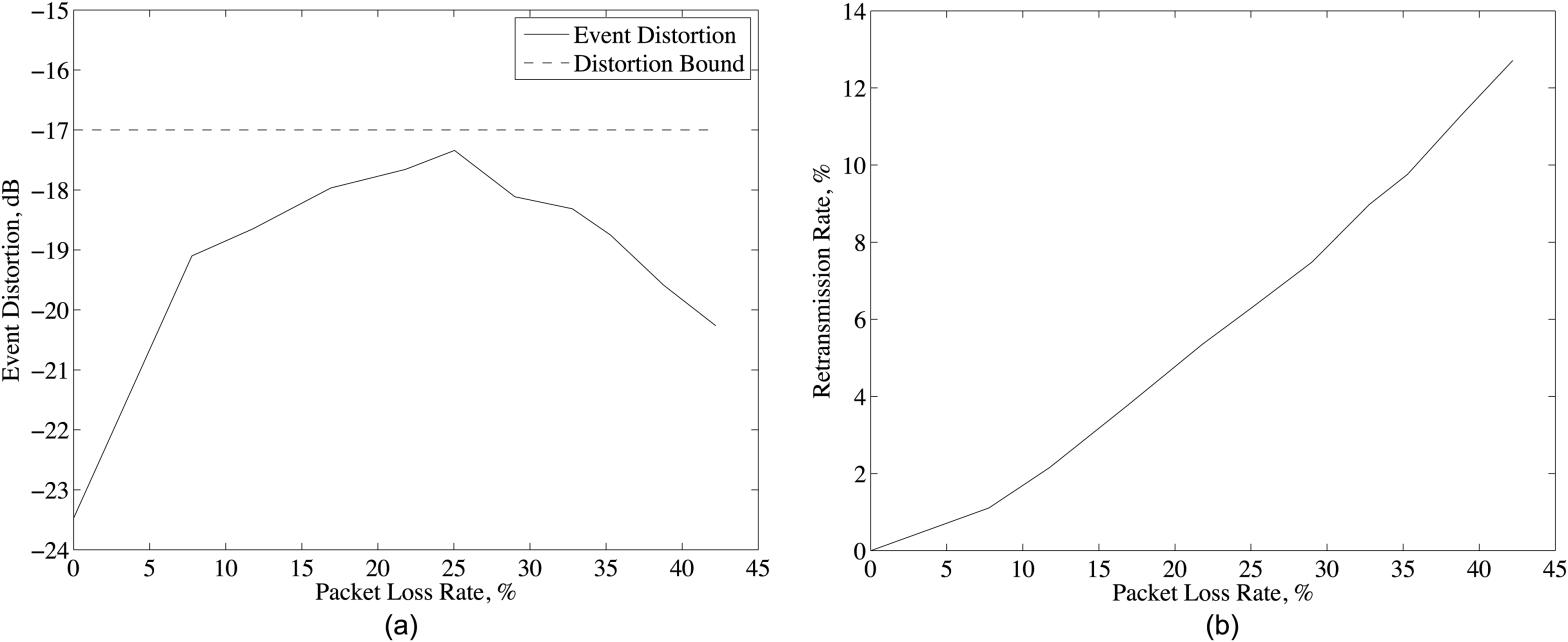
(a) Event distortion and (b) packet retransmission rate for different packet loss rates and system parameters given in Table 1 with *ρ*_*th*_ = 0 for music record.

As in Fig 11a, event distortion increases with packet loss rate and then, decreases before reaching desired event distortion *D*_*max*_. This is mainly related to retransmission rate of the packets as in Fig 11b. For low packet loss rates, D-DSC requests small number of retransmissions and let the event distortion to increase. However, further increase in packet loss rate increases event distortion above *D*_*max*_, then D-DSC increases the requests for retransmission to reduce the event distortion again below *D*_*max*_. Thus, event distortion always stays below event distortion bound. Fig 11b shows that the retransmission of at most 25% of the lost packets is enough to achieve the desired event distortion. Hence, D-DSC achieves the desired event distortion with the minimum number of retransmission regardless of packet loss rate.

## 5 Conclusion

D-DSC is a novel and unified approach that significantly improves the classical DSC by introducing a decoding delay concept for efficient data compression technique. With decoding delay, maximum correlated portion of sensor samples is used during the estimation of event features, and thus, power consumption is minimized. D-DSC has not only an efficient data compression technique but also an energy efficient and reliable protocol specification for event communication and estimation applications in WSN. Performance evaluation results show that D-DSC achieves reliable event communication and estimation for a practical signal detection/estimation application in sensor networks. It is observed that decoding delay concept can provide 10 times better compression rate than the classical DSC algorithms. Therefore, the proposed technique has potential to improve the performance of future IoST systems, where massive number of sensing and computing elements are deployed in order to interact and optimize the physical world.

## Supporting information

S1 Appendix Proof of (6) and (14). (PDF)
