# Supplementary material for: D-DSC: Decoding Delay-based Distributed Source Coding for Internet of Sensing Things
Source: PLoS One. 2018 Mar 14;13(3):e0193154. doi: 10.1371/journal.pone.0193154 (PMC5851590; doi:10.1371/journal.pone.0193154)
Supplement: S1 Appendix — (PDF) [file pone.0193154.s001.pdf]

D-DSC: Decoding Delay-based Distributed Source Coding for Internet of Sensing Things

S1 Appendix

Metin Aktas[1‡], Murat Kuscu[2‡], Ergin Dinc[2‡*], Ozgur B. Akan[2,3]

1 Aselsan, Inc., 06370, Ankara, Turkey

2 Electrical Engineering Division, Department of Engineering, University of Cambridge, CB3 0FA, Cambridge, UK

3 Deparment of Electrical and Electronics Engineering, Koc University, 34450, Istanbul, Turkey

‡These authors contributed equally to this work.

Proof of (6)

Here, we prove that (6) gives the minimum difference between samples of sensors i and j in MSE sense. For the samples with zero mean, variance of difference between sensor samples can be written as,

$$\begin{aligned} J(\xi, m) &= E\left\{e^2(n)\right\} \\ &= E\left\{x_i^2(n) + \xi^2 x_j^2(n-m) - 2\xi x_i(n) x_j(n-m)\right\} \\ &= \sigma_i^2 + \xi^2\sigma_j^2 - 2\xi R_{i,j}(m) \end{aligned} \tag{S1}$$

The minimum MSE can be found by minimizing (S1) with respect to both ξ and m jointly, which is

$$J_{min} = \min_{\xi, m} J(\xi, m) \tag{S2}$$

Since ξ and variances, σ_i^2 and σ_j^2 are positive values, (S2) can be simplified as,

$$J_{min} = \min_{\xi > 0} \left[\sigma_i^2 + \xi^2\sigma_j^2 - 2\xi R_{i,j}(d_{i,j})\right] \tag{S3}$$

where $d_{i,j}$ is the time difference between the maximum correlated data portion of node i and j given

in (8). The optimum value for ξ can be found by differentiating (S3).

$$\frac{\partial J_{\xi,d_{i,j}}}{\partial \xi} = 2\xi\sigma_j^2 - 2R_{i,j}(d_{i,j}) = 0$$

$$\xi = \frac{R_{i,j}(d_{i,j})}{\sigma_j^2} \tag{S4}$$

With minimum difference between samples of nodes i and j as in (6), the minimum variance is found as

$$J_{min} = \sigma_i^2 - \frac{R_{i,j}^2(d_{i,j})}{\sigma_j^2} \tag{S5}$$

Proof of (14)

From (11) and (13), correlation coefficient is found as $\rho_{i,j} = e^{-\frac{d_{i,j}^2}{\sigma}}$. Considering the clustering conditions (12), the maximum time difference between the samples of the master and slave nodes in one cluster is written as

$$d_{max} = min\left\{ d_{th}, \frac{1}{f_s} d_{i,j} \bigg|_{e^{-\frac{d_{i,j}^2}{\sigma}} = \rho_{th}} \right\} \tag{S6}$$

For sufficiently large d_{th}, (S6) can be simplified as

$$d_{max} = \frac{1}{f_s}\sqrt{-\sigma \ln \rho_{th}} \tag{S7}$$

Assuming that all sensors encode data with the same number of bits in (10), cost function in (13) is rewritten as

$$E_{tot} = E\left[(M-K)\log_2\left((2^{n_u}-1)\sqrt{1-\rho_{th}^2}+1\right) + Kn_u\right] \tag{S8}$$

where $M = \sum_{j=1}^{K} M_j$ is the total number of sensor nodes in the event area and K is the number of clusters. The maximum distance between master node and slave nodes in one cluster is $r_{max} = d_{max}v_s$, where v_s is the propagation speed of the source. Since we assume that sensor nodes are uniformly randomly deployed on the area of size $[r_e, r_e]$, the number of sensor nodes on the area of size $[r_{max}, r_{max}]$ can be approximated as,

$$M_j \cong \frac{r_{max}^2}{r_e^2} M \cong \frac{-\sigma \ln \rho_{th} v_s^2}{f_s^2 r_e^2} M \tag{S9}$$

Therefore, the number of clusters is found to be

$$K = \frac{M}{M_j} \cong \frac{f_s^2 r_e^2}{-\sigma \ln \rho_{th} v_s^2} \tag{S10}$$

Substituting (S10) into (S8) yields the cost function in (14). Since the number of clusters is limited with the total number of sensors, M, it restricts the selection of ρ_{th} as follows

$$\begin{gathered}
1 \leq K \leq M \\
\frac{1}{M} \leq \frac{-\sigma \ln \rho_{th} v_s^2}{f_s^2 r_e^2} \leq 1 \\
\frac{f_s^2 r_e^2}{\sigma v_s^2} \leq \rho_{th} \leq \frac{f_s^2 r_e^2}{-\sigma v_s^2 M} \\
e^{-\frac{f_s^2 r_e^2}{\sigma v_s^2}} \leq \rho_{th} \leq e^{-\frac{f_s^2 r_e^2}{\sigma v_s^2 M}}
\end{gathered}$$
